# Optimizing reproductive performance in pangasius catfish broodstock: A review of dietary and molecular strategies

**DOI:** 10.1016/j.vas.2024.100375

**Published:** 2024-06-16

**Authors:** Donald Torsabo, Sairatul Dahlianis Ishak, Noordiyana Mat Noordin, Khor Waiho, Ivan Chong Chu Koh, Muhammad Abduh Yazed, Ambok Bolong Abol-Munafi

**Affiliations:** a Higher Institution Centre of Excellence (HICoE), Institute of Tropical Aquaculture and Fisheries, Universiti Malaysia Terengganu, Kuala Nerus, Terengganu, Malaysia; b Department of Fisheries and Aquaculture, Joseph Sarwuan Tarka University, Makurdi, Makurdi, Benue State, Nigeria; c Faculty of Fisheries and Food Science Universiti Malaysia Terengganu, Kuala Nerus, Terengganu, Malaysia; d Guangxi Key Laboratory of Beibu Gulf Marine Biodiversity and Conservation, College of Marine Sciences, Beibu Gulf University, Guangxi, China; e Center for Chemical Biology, Universiti Sains Malaysia, Bayan Lepas, Penang, Malaysia

**Keywords:** Pangasius broodstock, Nutrient manipulation, Reproductive performance, Genomics, Proteomics

## Abstract

*Pangasius* catfish, a significant player in the global whitefish market, encounters challenges in aquaculture production sustainability. Quality broodstock maintenance and seed production are impeded by growth, maturation, and fecundity issues. This review investigates the efficacy of strategic nutrient composition and molecular strategies in enhancing broodstock conditions and reproductive performance across various fish species. A notable knowledge gap for *Pangasius* catfish hampers aquaculture progress. The review assesses nutrient manipulation's impact on reproductive physiology, emphasizing *pangasius* broodstock. A systematic review analysis following PRISMA guidelines was conducted to identify research trends and hotspots quantitatively, revealing a focus on *P. bocourti* and fertilization techniques. Addressing this gap, the review offers insights into dietary nutrients manipulation and genetic tool utilization for improved seed production, contributing to *pangasius* catfish aquaculture sustainability.

## Highlights

• Pangasius has substantially contributed to the international global supply of whitefish market.
• The current knowledge on dietary nutrient manipulation in fish diets to improve reproductive function and seed production.
• Effects of various nutrient groups were evaluated on general reproductive physiology and nutrient manipulation in bloodstock diets impacted reproductive capacity.
• Molecular techniques such as genomics, proteomics, transcriptomics, and metabolomics is gaining traction in all fields of aquaculture, including nutrition.

## 1 Introduction

**Fig. 1:**
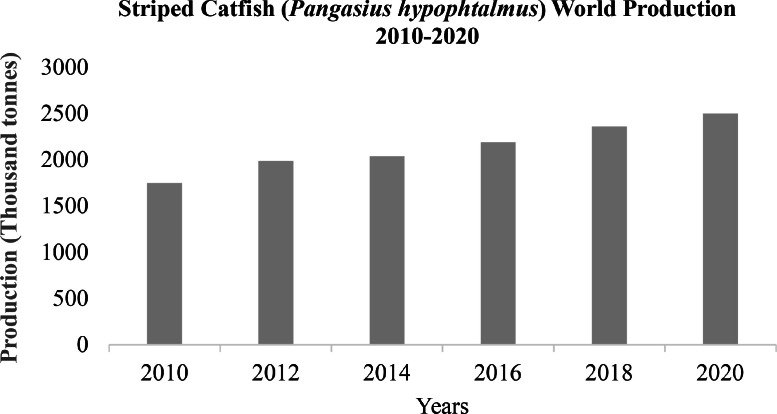
The worldwide production of striped catfish, *Pangasius hyphophthalmus*, from years 2010 to 2020. Source from FAO 2020; Zannat et el., 2023.

*Pangasiids* catfish can be found in major freshwater rivers of South Asia to Southeast Asia. For several decades, this type of catfish has been particularly significant for fisheries in several Asian countries (Gustiano et al., 2021). *Pangasius hypophthalmus, Pangasius boucorti* and *Pangasius djambal* are the three species most cultured in Southeast Asian aquaculture (Legendre et al., 2000; Gustiano et al., 2021). Other species from the family *Pangasidae* such as *Pangasius nasutus* and *Pangasius kunyit* are considered of great potential for aquaculture when breeding programs are established (Gustiano et al., 2021). Food and Agriculture Organization of the United Nations (FAO, 2020) reported that the total aquaculture production comprised 87.5 million tonnes of aquatic animals mostly for use as human food, 35.1 million tonnes of algae for both food and non-food uses, 700 tonnes of shells and pearls for ornamental use, reaching a total of 122.6 million tonnes in live weight in 2020. In 2020, farmed finfish reached 57.5 million tonnes (USD 146.1 billion), including 49.1 million tonnes (USD 109.8 billion from inland aquaculture and 8.3 million tonnes (USD 36.2 billion) from mariculture in the sea and coastal aquaculture on the shore (Naylor et al., 2021). Vietnam's *Pangasius* catfish production contributes 4.3% to this value with other Asian countries that are also major aquaculture producers including China, Bangladesh, Indonesia, and the rest of Asia (FAO, 2020). In recent years, *pangasius* has substantially contributed to the international whitefish market. The global supply of whitefish in 2016 was 15.4 million tonnes, with farmed *pangasius* making up 14% of the total supply (Thong et al., 2020). Fig. 1 showcases the steady increase of the striped catfish (*P. hyphophthalmus*) world production from 1749.4 thousand metric tonnes in 2010 to 2500.5 thousand metric tonnes in 2020 (FAO, 2020). Vietnam is the largest producer of *pangasius* catfish in the world with a production of 1.3 million tonnes. The global export value of *pangasius* in 2018 was $2.26 billion, with Vietnam representing over 90% of the global export value (Thong et al., 2020). As a result, *pangasius* aquaculture provides a significant portion of the protein supply. The demand for *Pangasius* catfish is not only limited to the whole fish and fillets but also to its various value-added products such as surimi, pickles, sausages, and noodles (Rathod et al., 2018). Similarly, the fish is also exploited for fish protein hydrolysate (FPH), hydroxyapatite (HA), and lipid fraction (Halim et al., 2016; Nam et al., 2020). Consistently, Gelatin produced from fish skin during fillet processing is used in the production of edible films because of its low melting point, low oxygen permeability, and better film-forming ability (Nurdiani et al., 2023). The increased aquaculture production of the *P. hypothalamus* is predicated on certain desirable characteristics such as fast growth, high survival rates, high market demand, its ability to withstand high stocking density in ponds, and its large size at harvest (Ali et al., 2018; Okomoda et al., 2020).

A major setback in the culture of *pangasius* has remained breeding in captivity, which is related to late sexual maturity and improvement in mass production. These setbacks can be addressed by improving the broodstock condition in terms of growth, maturation, egg quality, sperm quality, and fingerling mass production through the manipulation of nutrients in their diets (Izquierdo et al., 2015).

In general, good aquaculture production depends on feeds, which are the most important part of aquaculture. Poor-quality feed can significantly reduce the production rate. Thus, the use of high-quality feeds and proper nutrient combination leads to fast growth, enhanced reproductive performance, and good survival rate of fingerlings (Soltan,2009; Izquierdo et al., 2001). Macro and micro nutrients (protein, lipids, carbohydrates, vitamins, and minerals) combined in the right proportion in fish broodstock diets have been reported to influence broodstock fertility, and fecundity, which entail gonad maturation, egg, and sperm viability, hatching, and larval growth (Izquierdo et al., 2001). The quality and size of the fish eggs are important factors for successful seed production as larger fish eggs will eventually produce larger offspring and consequently have a physical advantage in their growth and survival due to efficient prey acquisition and environmental tolerance (Zakeri et al., 2009).

Protein through the supply of adequate amino acids has been found to enhance the reproductive performance of various freshwater and marine fishes (Finn & Fyhn, 2010; Kabir et al., 2015; Jobling, 2016). Lipid manipulation in broodstock diets of several fish species has proven to be of effect in promoting reproductive performance in freshwater finfish species as reviewed by Torsabo et al. (2022). Lipid movement from both liver and muscle of white seabream, *Diplodus sargus* to its gonads during gonadogenesis (Perez et al., 2007), and FA composition changes in gonad tissues of rainbow trout, *Oncorhynchus mykiss* broodstock (Mustafa et al., 2020), suggest that dietary lipid intake affects broodstock lipid composition and subsequently dictates fish reproductive maturation and gonadogenesis processes. Consistently, an increase of egg size and lipid droplets in the ovary histology, mirrored lipid droplets decreased in muscle sections of Chinese sturgeon (*Acipenser sinensis*), which suggests the consumption of muscle energy/lipid reservoir during ovarian development from stage II to IV (Leng et al., 2019). Carbohydrates’ specific roles in promoting reproductive performances in fish have not been clearly defined, though they play an important role as a lower-cost of energy source in comparison to dietary protein. Micronutrients (vitamins and minerals) perform important functions in fish physiology, which include cellular respiration, enzymatic activities, wound healing, oxygen transport, protein stability, free radical scavenging, and protection from skeletal deformities (Henry et al., 2020). As a result, the nutritional components play a critical role in the reproduction process and the creation of high-quality eggs and offspring, which will boost gross fish output and cut down the dependency on wild-harvested seed (Izquierdo et al., 2001; Watanabe & Vasallo-Agius, 2003; Migaud et al., 2013).

The application of molecular techniques in aquaculture, including genomics, proteomics, transcriptomics, and metabolomics, is gaining traction. These techniques have been successfully applied in various areas of aquaculture, such as hatchery production, nutrition, disease and immunology, and post-harvest quality control (Alfaro & Young, 2018; Bernatchez et al., 2017). Metabolomics, in particular, has shown potential in solving aquaculture problems and is being increasingly used in research (Li & Wang, 2017). Despite the potential, the application of genomics in aquaculture and fisheries is still underdeveloped, with a need for improved research and industrial applications (Cancela et al., 2010). However, the use of genomics in aquaculture is growing, with a focus on genetic improvement through selective breeding and the potential for further advancements in the future (Houston et al., 2020). This review aims to consider the current knowledge on dietary nutrient manipulation and molecular strategies employed over the years and how this approach has improved seed production of Pangasius catfish over time, as well as to describe the impacts of different dietary nutrient categories on the reproductive events of the fish family.

## 2 Systematic review analysis

A systematic search of literature related to research on Pangasius broodstock reproductive performance was conducted following Preferred Reporting Items for Systematic Reviews and Meta-Analyses (PRISMA) guidelines to identify the research hotspots and trends quantitatively (Chen & Song, 2019). The study applied the PRISMA criteria as a well-established standard for performing systematic literature search via Scopus and ISI Web of Science (WoS) Core collection. These two sites are major multidisciplinary scientific databases, with higher authenticity and extensively peer-reviewed. The search string was designed as (“pangasius” OR “pangasidae” OR “pangasiid” OR “pangasionodon”) AND (“reproductive” OR “reproduction”). A total of 29 articles were obtained from the initial screening of the peer-reviewed literature in the Scopus and WOS databases (search string within the titles, abstracts, and keywords). The search string only produced two document types: research and review articles, and no other document types such as conference proceeding, book chapters, letters and etc. were found. The number of documents were then reduced to 20 by eliminating duplicate articles found in both databases upon further screening. The next screening was based on the title of the articles, and articles that did not match the inclusion criteria were excluded with a total of 14 documents remianed.

VOS viewer software (version 1.6.20) was utilized to conduct the bibliometric analysis.

**Fig. 2:**
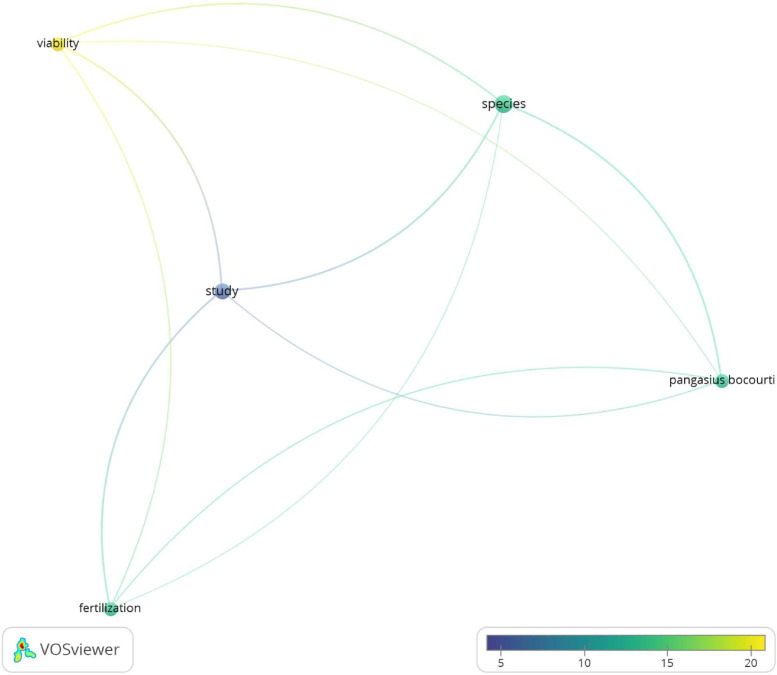
Co-occurrence keyword analysis according to cluster. The difference in colour indicates the average number of citation, where the size of the circle indicated the frequency of occurrences.

A keyword co-occurrence analysis was performed based on the keywords provided by the authors from these 14 documents, and the top 5 most frequent keywords (among 739 keywords) (Table 1). As shown in Table 1, the five most frequently used keywords are: species, study, *Pangasius bocourti*, fertilization, and viability. It indicates that the research on pangasius broodstock focuses more *P. bocourti* compared to other species. Other study focus are on fertilization techniques and viability of sperm. Fig. 2 shows the link strength between the keywords. It can be seen that there are a lot of research gaps to be filled on the improvement of Pangasius broodstock irregardless of species.

**Table 1:** The top 5 most frequently occurring keywords in these documents.

**Keyword**	**Occurences**	**Total link strength**
species	10	12
study	8	14
*Pangasius bocourti*	6	9
fertilization	6	9
viability	6	10

## 3 *Pangasius* catfish: taxonomy, biology, habitat and distribution

*Pangasiidae* are riverine catfishes that exit in freshwater habitat with enormous economic impotence. The *Pangasius* genus contains 21 recognised species. The species are found throughout Southeast Asia, from the Indonesian Archipelago up to India in the southern region of the Asian continent. All other species reside in the middle or upper part of the major rivers, except for *Pangasius sabahensis, P. mekongensis, P. kunyit, P. krempfi, and P. rheophilus,* which have distributions up to estuaries (Gustiano et al., 2018). *P. hyphophthalmus, P. boucorti,* and *P. djambal* are three of the twenty-one species of *Pangasius* catfish that have been extensively used in aquaculture production in Southeast Asia. Other potential species like *P. kunyit* and *P. nasutus* have also been identified pending when breeding programs are fully established (Gustiano et al., 2021). *Pangasius* catfishes are recognized by a laterally compressed body, with a pair of maxillary and mandibular barbels and relatively long anal and short dorsal fins with two spines and a small adipose fin with a free posterior margin (Gustiano et al., 2018). It is mainly known for its white and flaky boneless meat, which many use as a cheaper alternative to cod. *Pangasius* catfish is also commercially successful and contributes significantly to the worldwide whitefish market (Thong et al., 2020).

*Pangasius* catfish are mostly omnivores, consuming fruit, crustaceans, fish, algae, higher plants, and insects in addition to zooplankton, higher plants, and insects (Hogan, 2004; Poulsen et al., 2008). Among the family *Pangasidae, Yellowtail* catfish, *Pangasius pangasius* is considered carnivorous and voracious, and it preys on snails and other molluscs (Rahman et al., 2020). *Pangasius* catfish are divided into three groups considering their ratio of intestine length to standard length (li/ls): true filter feeders, omnivore/carnivores, and omnivore/filter feeders (Hogan, 2004; Razzaque et al., 2008). They inhabit large to medium freshwater rivers and tend to inhabit deep pools but can also be found in rapids and streams. During dry weather, the pangasius catfish find shelter in deeper refuge areas. Unlike the majority of the genus, *P. krempfi* spends part of its life in coastal waters. *P. hypophthalmus* and *P.bocourt* occur in large rivers and floodplains of Chao Phraya Mekong, while *P.Kunyit* and *P. rheophilus* are native to Indonesia*. P. nasutus* occurs in Sumatra, Indonesia and Peninsula Malaysia in Perak and Pahang rivers (Gustiano et al., 2018). Migratory movement is common to all pangasius catfishes for different purposes at different life stages. This migratory movement occurs in three forms, one from the flooded habitat to the main river channel during the rainy season, the second is the movement of adults up and down the main river channel, and thirdly, the migration of young fish from downstream (Hogan, 2004). For example, *P. pangasius* found in large rivers and estuaries, migrate as juveniles to brackish water and become sub-adults and during the adult stage they move to river mouths and inshore areas (Hogan, 2004). These important aquaculture species though not native to many countries, have been introduced for aquaculture purposes and are contributing immensely to aquaculture production worldwide.

### 3.1 Pangasius culture and production

*Pangasius* catfish aquaculture in the early years depended on the wild stock for the supply of seed, which has now been replaced by hatchery produced seed in most countries, especially the principal producer Vietnam as a result of hatchery development and technological advances (Brugere et al., 2021; Hasan et al., 2021). However, several producing countries to some extent still depend on wild-captured juveniles to stock ponds as they face difficulties to maintain consistent seed production from broodstocks in captivity (Nguyen et al., 2013). Matured *pangasius* broodstock in captivity is induced with human chorionic gonadotropin or pituitary gland extract to spawn. The hormone is injected between 4-5 doses at a time in the female broodstock, (at average doses of 542, 597, 893, and 3442 IU kg-1 or 500, 500, 500, 1500, and 3000 IU kg-1 at time intervals of 0, 23, 46, 56 and 66 h), ovulation takes place in 5-11 h after the last injection at 28-29°C (De Silva & Phuong, 2011). The eggs are dry stripped and mixed with the sperm and incubated in stainless steel or glass with up-welling water flow to keep the eggs in suspension. Eggs usually hatch within 22-24 hours and it takes another 24 hours for larval yolk absorption before larvae are transferred to nursery ponds (Kikenge,2022). The nursery period is divided into two stages characterized by the type of feed. In the first nursing period from larvae to 0.3-1g fry, mixed zooplankton live *Moina* or artemia is used and in some instances, soybean meal and cooked egg yolk are blended to form an emulsion and fed to fry. The feeding rates and frequency are different among farmers, ranging from 5-18% per biomass daily, and 4-8 times per day (De Silva & Phuong, 2011; Sahoo & Ferosekhan, 2018). The second stage of nursing from fry to fingerlings 14-20g for two months is categorized with the introduction of commercial pelleted feed. For example, the ICAR-Central Institute of Freshwater Aquaculture has developed larval feed starter-p for pangasius catfish larvae (Sahoo & Ferosekhan, 2018). About 96% of nursery farmers prefer commercial feeds to farm-made feeds because they are simple to handle, utilize, and store, have higher quality, and are more effective. For example, farmers that used farm-made feed recorded a high mortality rate (52 ± 10%) at a stocking density of 497 ± 188 fish m^−2^ compared to farmers that did not use farm-made feed with a mortality rate of 29 ± 6% at a stocking density of 154 ± 55 fish m^−2^ from fry-fingerlings stage (Bui et al., 2010). The fingerlings are moved to grow-out locations, such as earthen ponds, net cages, and net pens. *Pangasius sp.* can be stocked at high densities of 120m^2^ and tolerates foul water with dissolved oxygen levels as low as 0.05-0.10 mg L^−1.^ The growth out face of tra catfish in the Mekong Delta adopted the pond culture system with pond depth ranges from 2.0 -6.0 m to prevent the escape of fish during flood season into the main river. The stocking density of several farms ranges from 18- 125 fish m^−2^ to 5-31 fish m^−3^ depending on the size and availability of seed and the financial status of the farmers (De Silva &Phuong, 2011). Depending on the growth conditions, most cultured *pangasidae* families achieve 1.0-1.5 kg after six months or less (Phu & Hein, 2003).

### 3.2 Pangasius reproduction and biology

*Pangasiid* catfish are potamodromous migratory fish that travel hundreds of kilometers between upstream refuge and spawning sites and downstream feeding and nursery habitats. Adult fish can reach a maximum length and weight of 130 cm and 44 kg, respectively. Most of the species are considered benthopelagic, living within pH ranges of 6.5 – 7.5 and temperatures ranging within 22-26℃. Males mature in their second year in captivity, while Sexual maturity in females takes at least three to four years in captivity, which is probably the same time in the wild as maturity in the wild has not been reported. A mature female of up to 10 kg in weight can spawn above one million eggs (Kibenge,2022). Broodstock in the wild has been known to spawn twice a year, however, in ponds and cages, it has been observed to spawn again after 6–17 weeks from the initial spawn (Kibenge, 2022). Spawning takes place from May to June when the monsoon season begins, and during the wet season, they migrate to deeper waters for refuge. Spawning grounds consist of rapids and sandbars interspersed with deep rocky channels and pools. The eggs are sticky and are deposited in the exposed root system of several aquatic tree species (Phuong & Oanh, 2010). *P. bocourti* catfish is a fish species that exhibits a secondary sexual characteristic. To determine the sex of the fish, a flexible catheter is needed. The natural sex ratio of male and female fish is 1:2. The fish reaches 50% maturity at a length of 9cm in the Mekong River (Kulabtong, 2012). The fish spawns upstream in the Mekong River and its large tributaries in Thailand from April to June. According to Poulsen et al. (2004), *bocourti* catfish lays eggs in the Mekong mainstream and the larvae drift downstream to the floodplain area. This area provides a suitable nursery habitat for larvae. After the flood season, the juvenile fish migrate downstream to the lower Mekong River, where they spend the dry season (Poulsen et al., 2004). Turbid water triggers the spawning behavior and the larvae appear in June. However, in aquaculture, the fish can spawn throughout the year. The eggs of bocourti catfish are yellow and round-shaped, with an average diameter of 1.28mm. The eggs are demersal and sticky. The fecundity of bocourti catfish varies according to different reports ranging from 6,980–9,563 eggs/kg BW, 21,139 eggs/kg BW, and 157,040 eggs for female spawners weighing 5.2–12.2kg BW (Kulabtong, 2012).

## 4 Nutrition, feed, and feeding in pangasius culture

Nutrition is an important factor in the reproductive success of all fish species, and so proper diet and nutrition will have a significant impact on any farmed fish's ability to maximise its genetic potential for enhanced growth, reproduction, and survival. (NRC, 2011; Berlinsky et al., 2020). Fish require energy to maintain basic metabolic functions as well as to promote growth, reproduction, mobility, and health (Turchini et al., 2019). Macronutrients are obtained from proteins, carbohydrates, and lipids which are essential for energy production while micronutrients, such as vitamins and minerals, are not energy-related nutrients; rather, they function as metabolic pathway precursors and are essential for good performance (De Silva & Anderson, 1995; NRC, 2011; Hamre et al., 2011). Fish require essential amino acids and proteins for development, tissue repair, general health, and reproduction (Herring et al., 2021). The amount and sort of amino acids in a protein source decide the general protein quality (Hernandez de-Dios et al., 2022). Fish meal, for example, is a high-quality protein but is considered costly (Turchini et al., 2019). Fish meals as a source of protein can be replaced with less expensive protein sources, provided they fulfill the essential amino acid requirement of the fish (Nandi et al., 2023). Lipids are high in energy and contain essential fatty acids (n-3 and n-6) as well as fat-soluble vitamins (A, D, E, and K) that are necessary for optimal fish development, health, and reproduction (Dong et al., 2023). Phospholipids such as soybean lecithin are often needed by crustaceans, molluscs, and sometimes young fish (Nakamura, 2017). Fatty acid requirements vary by species: herbivorous and omnivorous fish thrive on plant oils that contain shorter-chain fatty acids, but carnivorous fish require longer-chain fatty acids such as those found in marine fish oil (Luo et al., 2023).

Vitamins and minerals, which are organic and inorganic components that are required in small amounts for normal growth, health, and general function of the fish body, make up micronutrients. Micronutrients perform important functions in fish physiology, which include cellular respiration, enzymatic activities, wound healing, oxygen transport, protein stability, free radical scavenging, and protection from skeletal deformities (Henry et al., 2020). Deficiency of micronutrients in fish diets can result in low feed utilization, impaired growth, and diseases in cultured fish (Oliva-Teles, 2012; Henry et al., 2020).

### 4.1 Types of feed

Different feed types are utilised in *pangasius* catfish production which includes fresh feed, formulated feed (artificial diets), and mixed (a blend of fresh and artificial) feeds. Fresh feeds are made from trash fish and other agricultural waste which are fed directly to the fish while some farmers locally process and produced them as sinking feeds at the farm site (Nguyen, 2013). Fresh feeds are easy to access and relatively cheap compared to formulated feeds although their storage is meant to be for a shorter period due to their high moisture content. It also presents a challenge in terms of water quality management as they are fresh and easily sinks to the bottom of the culture facility leading to water quality deterioration and an increase in disease outbreaks in farms (Nandeesha et al., 2013). On the other hand, formulated feeds are easy to store as they contain less moisture but are expensive for local fish farmers to purchase for daily feeding on farms. Formulated feeds are made from a variety of feed ingredients mixed in a specified ratio to satisfy the nutritional needs of fish. Formulated feeds come in pelleted form and can float in a variety of sizes depending on the fish's life stage (Nguyen, 2013). The floatability of formulated feed is an advantage in feeding practice as it allows for improved feed utilization, reduces disease incidents, and serves as a medium to regulate overfeeding which could result in water quality deterioration (Nandeesha et al., 2013). Some *pangasius* farmers feed their broodstock on a combination of fresh and formulated diets (Bui et al., 2010).

### 4.2 Feed Formulation

Feed formulation is the process of calculating the volume of ingredients and additives to blend to make compound feeds that match the known nutrient requirements of the targeted species while also meeting production goals at a low cost (Lall & Dumas, 2015). *Pangasius* farmers used farm-made feeds produced from waste fish, soybean meal, broken rice, rice bran, blood meal, cotton seed flour, milk, eggs, and vegetables (such as green peas and water spinach) in the 1990s (Bui et al., 2010). The ingredients are mixed and supplemented with vitamin C and E premixes, cooked, and fed to the fish as semi-moist feed subjected to an extruder to produce pellets.

Commercial pelleted feeds have now been made to be available through the knowledge of feed formulation and technological advances in the feed industries. Commercial pellets are becoming more popular due to concerns over food safety and the inconsistent quality of farm-made feed. However, the commercial feeds made available for *pangasius* catfish culture did not include those for broodstock. Generally, the levels of inclusion of certain ingredients in fish diets change with the life stages of the fish, for example, the protein requirement for fry or fingerlings will not be the same for broodstock due to different activities at various stages of life (Izquierdo et al., 2001).

Broodstock diets have been formulated for *P. hypopthalmus*. Kabir et al. (2019) formulated diets containing 30 % protein (soybean meal and fish meal) and 9-12 % lipids (fish oil and crude palm oil) which performed significantly well on broodstock performance in terms of fecundity, fertilization, spawning, and egg quality compared to some commercial diets with 22-26% protein, 4-6 % lipids, and 28.0% protein, 5.0% lipids (Rahman, et al., 2020). These commercial diets are general grow-out feeds that are being used for feeding broodstock instead of formulating diets to meet *pangasius* broodstock nutrient requirements. The use of normal grow-out feeds for broodstock might have constituted the poor performance of brooders, considering the demand for nutrient requirements for breeding activities. It is, therefore, necessary to formulate feeds for the broodstock of *Pangasius* catfish that will adequately meet the demand for various nutrients required for pre-spawning and spawning activities. *Pangasius* feeds are currently produced from a wide range of locally and imported ingredients. Common feed ingredients used in pangasius catfish production around the globe are summarized in Table 2. The distribution of ingredients across different locations varies as some particular ingredients might be lacking in certain locations, leaving the farmers the choice to use available ingredients in their location depending on ingredient nutrient contents.

**Table 2:** Some commonly used feed ingredients in pangasius catfish production around the globe.

Major ingredients	Other ingredients	Minerals/Vitamins/additives
Fish meal	Egg	Methionine
Broken rice	Cassava	Mineral
Rice bran	Catfish extract oil	Premix
Soybean meal	Marine trash fish	Pre-biotic (glucan)
Soybean cake	Crude palm oil	Probiotic
Wheat flour	Canola meal	Sorbitol
Mustard oil cake	Blood meal,	Vitamin C
Meat and bone meal	Freshwater fish oil	Calcium
Dry fish	Vegetable oil	Growth promoter
Rice polish	Corn starch	Feed binder
Corn flour	Cotton seed meal	Lysine
		Pangarenol
		Pozyme
		Vimidime
		Vitex
		Vemedin

The ingredients are summarized from the formulations of Debnath et al., 2005, Tran et al., (2019); Kabir et al., (2019), Sattang et al., (2021), and from a review by Haque et al. (2021)

## 5 Pangasius broodstock nutrition

In most cases, the reproductive axis is hampered by negative energy balance and insufficient food intake, making it difficult to maintain the high-energy demands of gametes and reproductive processes (Seifi Berenjestanaki et al., 2014; Laining et al., 2019). During the period of gonad maturation, the energy demand for most species of catfish is increased, especially during vitellogenesis. As the embryo depends on the egg yolk until the larvae are ready to feed externally, the quality of the egg is affected by the quantity of the egg yolk. (Hariani & Pungky, 2019). Adequate feeding is essential, especially for newly domesticated broodstock fish harvested for breeding in captivity. In the *Pangasius* fish, high stress tolerance and the ability to acclimate e to a new environment become important limiting factors influencing reproductive performance (Asdari et al., 2011). Farmers of *pangasius* catfish have taken advantage of the omnivore nature of some species such as *P. hypophthalmus* by feeding their broodstock with several local ingredients such as cooked de-oiled rice bran (DOB), broken rice, and discarded potatoes from agricultural waste (Lakra, 2010). The practice of feeding broodstock of *pangasius* with feed lacking the required nutrients might have been the reason for poor broodstock performance resulting in hatchery operators keeping more broodstock to achieve seed production. Broodstock performance has been enhanced through nutrient manipulation in several fish species, an area that has been given less attention in the family *Pangasidae*. Lipids (fatty acids) and proteins (amino acids) in broodstock diets have been established to be the main dietary variables that determine good reproductive efficiency (Izquierdo et al. 2001). For instance, increased dietary protein levels in Nile tilapia (*Oreochromis niloticus*) broodstock diets from 30% to 40% resulted in increased spawning performances, including the total number of spawning per tank, number of spawning per female, egg diameter, oocyte diameter, absolute fecundity, and average number of eggs per spawn, which were all significantly higher (P˂0.05) at 40% CP than at 30% protein levels (El-Sayed & Kawanna, 2008) also Nile tilapia receiving 45% dietary protein spawned more frequently than those receiving 25% dietary protein (Siddiqui et al., 2019). The increased nutritional deposition in the yolk was related to the dietary protein levels, which will affect the oocyte and egg diameter. Directly enriching the diet of channel catfish (*Ictalurus punctatus*) with a lipid topcoat rich in highly unsaturated fatty acids (HUFAs) greatly impacts the quality of egg and brooders efficiency (Durland, et al., 2010).

The nutrient requirement of embryonic and larval stages can only be met if the right biochemical composition is made available to the brooders as dietary nutrients from maternal reserves are mobilized into the eggs during exogenous feeding (Brooks et al., 1997; Watanabe & Vassallo-Agius 2003; Mejri et al., 2017). Improving the nutritional composition of broodstock diet has a significant effect on egg and sperm efficiency, as well as larval longevity (Izquierdo et al., 2001). Certain basic nutritional supplements alter gonadal development and fecundity in a few fish species, particularly in ceaseless spawners with short vitellogenic periods (Jobling, 2016). Female health is mainly influenced by their diet; therefore, a healthy diet is essential for enhanced reproductive success. In a species-specific manner, fatty acid ratios (EPA/ARA/DHA, n-3/n-6) directly influence the composition and content of eggs (Furuita et al.,2007). Therefore, the ratio of n-3/n-6 fatty acids in broodstock diet must also be considered (Ng & Wang, 2011; Hilbig et al., 2019). Some research on the content of n-6/n-3 fatty acids that resulted in the highest growth and hatching rates have been undertaken. According to Utiah et al., (2007) n-6 fatty acid content of 1.56% and n-3 fatty acid content of 0.78% produced 68.2% broodstock fecundity with the highest hatching rate of 90%. Waspada (2012) stated that the average value of egg fecundity per kilogram ranged from 11,589-15,802 eggs/kg in striped catfish, *P. hypopthalmus* broodstock which were given different kroto flour with crude fat content (3.35%) could increase egg fecundity. Fecundity is influenced by fatty acid levels in the feed, especially n-3/n-6 fatty acids, which affect reproductive performance.

The rate of feeding also affects the growth and development of broodstock. According to Estrada-Godinez et al. (2021) *P.hypopthalmus* broodstock fed at 1%, 2%, and 3% biomass showed significant differences in their growth and spawning performance, with those fed at 3% outperforming those fed at 2% biomass in terms of growth and spawning performance while those fed at 1% biomass show no spawning activity. There is more that can be done with pangasius catfish with similar applications to improve their reproductive performance considering the numerous successful reports that have been highlighted in other works (Utiah et al., 2007; Jobling, 2016; Hilbig et al., 2019; Estrada-Godinez et al., 2021) regarding dietary nutrients and their influence on several reproductive parameters of some freshwater and marine fish species.

### 5.1 Proteins and amino acids

Proteins are large complex molecules made up of hundreds or thousands of smaller units known as amino acids essential for the structure, function, and control of the body's tissues and organs (Kuhn, 2017). Proteins in an organism's body are classified according to their roles, which include antibodies, enzymes, messengers, transport/storage, and structural components (Blanco and Blanco, 2017). In aquaculture, several ingredients have been combined in the feed formulation process to supply the needed protein required by various fish species for different functions in their body, one such ingredient is fish meal (Hua et al., 2019).

Fish meal is preferred for use in aquafeed because of its amino acid composition which perfectly meets the requirement of both freshwater and marine fishes for proper body function (Frochlich et al., 2018). The most reliable source of protein for the aquaculture feed industries is considered to be fish meal made from small pelagic fishes, but due to the declining availability of wild fish for feed production and the ongoing rise in the price of feed for aquaculture, it has become necessary to use alternative sources of protein in aquafeed. (Shepherd and Jackson, 2013; Hua et al., 2019). As alternative protein sources to fish meal in aquafeeds, a few plant protein components (e.g., soybean meal, maize gluten meal, rapeseed meal) and animal by-products (e.g., meat and bone meal, poultry meal) are being used. While these terrestrial plant-based proteins (such as soy concentrate) will continue to be important components of aquafeeds, they have several drawbacks, including fatty acid profile, amino acid profile, mineral profile, palatability, mycotoxin content, and the presence of anti-nutritional elements (Malcorps et al., 2019). These proteins obtained from different sources have been used for feed formulation in aquaculture by various feed companies and researchers for the culture of aquatic animals looking at different targets such as growth, reproduction, and several other parameters (Turchini et al., 2019). Protein through the supply of adequate amino acids has been found to enhance the reproductive performance of various freshwater and marine fishes (Finn & Fyhn, 2010; Kabir et al., 2015; Jobling, 2016).

Protein and their amino acid affect reproductive performance in fish (Bobe & Labbe, 2010; Coldebella et al., 2011). Amino acids (AA) are required for larval growth as a metabolic fuel (Kuhn, 2017). Protein synthesis, growth, or both are known to be hampered by deficiencies or excesses of one or more amino acids (Bobe & Labbe, 2010; Lanes et al., 2012). Amino acids are vital in ovarian maturation because they cause modifications that result in a large amount of protein being incorporated into the oocytes, and vitellogenin is a key vehicle for delivering amino acids (Finn & Fyhn 2010; Edwards et al., 2019). Aside from normal tissue and organ development, amino acids are essential for fertilisation and embryonic development (Arukwe & Goksøyr, 2003).

An increase in fecundity, ovipositor diameter, and egg ripeness was reported by Kabir et al. (2015) in *P. hypophthalmus* fed varying levels of dietary protein from 250, 300, 350, and 400 g kg^−1^ in their diets. Fish fed with a diet containing 350 g kg^−1^ of protein showed significant differences among other groups. However, fish fed with 400 g kg^−1^ of protein in their diet showed no significant differences in terms of fecundity, ovipositor, and egg ripeness. This result was explained to be because of the rich nutrient content and all the essential amino acids present in the 350 kg^−1^ dietary protein for improved fecundity and egg ripening. Another reason could be that the optimal dietary protein requirement for *P. hypopthalmus* is 350 kg^−1^ and giving an excess of the optimum could not bring about any significant difference in the reproductive parameters examined. It would also be of interest to study the detrimental effects of dietary protein inclusion above the optimum requirements for *P. hypopthalmus*.

Consistently, the number of gametes produced by *P. hypophthalmus* females was influenced by the level of crude protein, according to Kabir et al. (2019). Females receiving meals containing around 338 g kg^−1^ protein had the highest absolute and relative fecundity. Similarly, the maturation time of gonads in green catfish (*Hemibagrus nemurus*) was reported to be faster with 37% dietary protein in their diets as well as increased fecundity and egg diameter (Aryani & Suharman, 2015). There is mounting proof that numerous AA regulates important metabolic processes that are essential for upkeep, growth, reproduction, and immunological responses in studies of both aquatic and terrestrial animals (Singh et al., 2021). Arginine plays a critical role in regulating endocrine and reproductive functions, as well as extra-endocrine signaling pathways and larval survival of fish. Survival of larvae from haddock broodstock-fed supplemented AA diet resulted in a 30% survival rate higher than the broodstock-fed basal diets (Li & Gatlin, 2006; Li et al., 2009). It is worth mentioning that from several findings, dietary protein tends to enhance the reproductive performance of different fish species at different inclusion rates, influencing several reproductive parameters such as gonad maturation, egg diameter, fecundity, total number of eggs per female, egg ripeness and ovipositor diameter, respectively. Studies on fish broodstock are currently scarce in the area of specific AA roles in promoting reproductive activities as compared to several studies reporting the roles of AA in fish larval and juvenile stages on growth, intestinal health, and other physiological-related parameters. Table 3 presents a summary of some commercial and experimental feeds with various levels of protein and lipids inclusion and their optimums and effects on different farmed fish species.

**Table 3:** A summary of feeds and the inclusion levels of macronutrients for reproductive performance of different farmed fish species.

Feed type	Species	Protein g kg^−1^		Lipid g kg^−1^		Remarks	Reference
		**Treatments**	**Optimum**	**Treatments**	**Optimum**		
Experimental	Striped catfish, *Pangasianodon hypophthalmu*s	250, 300, 350 and 400	350	90	90	350 &90 g kg^−1^ resulted in improved reproductive performance, 400, show no significance from 350, while 250 and 300 are reported inadequate for broodstock	Kabir et al., 2015
Commercial	Striped catfish, *Pangasianodon hypophthalmus*	350	350	70	70	The combination of protein and lipid fed at 3% biomass generated high fecundity in both sexes with high fertilization and hatching rates.	Estrada-Godinez et al. 2021
Experimental	Striped catfish*, Pangasianodon hypophthalmus*	300	300	60, 90, 120	90	The reproductive efficiency and egg quality of broodstock are significantly impacted by the lipid level at 90 g.kg-1 with 300 g.kg-1 protein in the diet.	Kabir et al., 2019
Experimental	Green Catfish *Hemibagrus nemurus Bagridae*	200, 270, 320, & 370	320 & 370	100	100	Gonadal maturation time, Ovi-somatic index, fecundity, egg diameter, and larvae production were all influenced by dietary protein levels.	Aryani & Suharman, 2015
Experimental	Snakehead murrel, *Channa striatus*	450	450	100, 140, & 180	180	Significant increases in GSI, fecundity, oocyte diameter, and number of mature oocytes were found in the group fed with diet containing 180 g kg^−1^ lipid level	Ghaedi et al., 2016
Experimental	Rainbow trout (*Oncorhynchus mykiss*)	423	423	20% (FO_80_/VO_20_), 50% (FO_50_/VO_50_), 75% (FO_25_/VO_75_) and 100% (VO_100_)	FO_25_/VO_75_	A blend of FO_25_/ VO_75_% improved reproductive performance. VO _100_% resulted in a low fertilization rate while FO_80_/VO_20_ showed a low survival rate for larvae	Noori et al., 2019
Experimental	Hybrid catfish (*Pangasius larnaudii* x *Pangasianodon hypophthalmus,*	300	300	0%, 1%, and 2% FFO	1%, and 2% FFO	The oocytes and spermatocytes from fish fed with 1% and 2% FFO were in higher histological stages of maturity, elevated levels of 17 β-estradiol and testosterone compared to the control.	Sattang et al., 2021

Abbreviations: FO, Fish oil, VO, Vegetable oil, FFO, Freshwater fish oil, GSI, Gonadosomatic inde

### 5.2 Lipids and fatty acids

Lipids are a set of fat-soluble substances present in plant and animal tissues that are broadly classified as fats, phospholipids, sphingomyelins, waxes, and sterols. Animals' principal energy stores are fats, which are fatty acid esters of glycerol used to meet long-term energy needs, such as during periods of intense exercise or when food and energy consumption are insufficient. Fish has the unusual capacity to quickly metabolise these substances and, as a result, can survive for long periods without food (Babalola & Apata, 2011; Turchini et al., 2011).

Fish oil has been used in aquaculture for decades to maintain normal growth, health, and nutritional quality of farmed aquatic animals because of its high quantity of n-3 long-chain polyunsaturated fatty acids (LC-PUFA) (Turchini et al., 2011). However, the demand for fish oil in aquaculture feed industries has continued to increase over time creating pressure on the wild stock because of over dependent on fish oil for feed production. Due to the decreasing nature of the popularly desired fish oil because of the sustainability and high cost related to it, alternatives lipid sources from terrestrial oils, especially vegetables have been given due consideration through several research works (Tocher, 2003; Turchini et al., 2010, 2011; Sankian et al., 2019). In comparison to fish oil, terrestrial oils are often rich in C18 fatty acids, primarily linoleic (LA, 18:2n-6), α-linolenic (ALA, 18:3n-3), and oleic (OA, 18:1n-9) acids, but lack or have a very low amount of n-3 LC-PUFA, such as docosahexaenoic (DHA, 22:6n-3) and eicosapentaenoic (EPA, 20:5-3) acids (Bureau and Meeker 2011; Nasopoulou & Zabetakis 2012). Depending on the species studied and the type and fatty acid concentration of the alternative oil utilized, literature suggests that most alternative oil sources can partially replace fish oil (Turchini et al. 2011). Terrestrial oil sources can be used in aquafeed for freshwater fish, as opposed to marine species, which appear to lack the ability to desaturate and elongate C18 PUFA and are therefore vulnerable to n-3 LC-PUFA deficit (Tocher 2010). Feeding studies on freshwater fishes such as the Malaysian mahseer, *Tor tambroides* (Bami et al., 2018; Kamarudin et al., 2018); silver catfish, *Rhamdia quelen* (Lazzari et al. 2016); Murray cod, *Maccullochella peelii* (Turchini et al. 2011); gibel carp, *Carassius auratus gibelio* (Zhou et al. 2016); pikeperch, *Sander lucioperca* (L.) (Kowalska et al. 2012); Nile tilapia, *Oreochromis niloticus* (Peng et al. 2015; Apraku et al. 2017); darkbarbel catfish, *Pelteobagrus vachelli* (Jiang et al. 2013); rainbow trout, *Oncorhynchus mykiss* (Gause and Trushenski 2013; Yildiz et al. 2018); and snakehead, *Channa striatus* (Aliyu-Paiko and Hashim, 2012); have shown that it is possible to combine terrestrial oils, and fish oils in adequate proportion to promote growth, reproduction and overall feed efficiency. Table 4 presents a summary of selected major aquaculture freshwater finfish species listed in the FAO (2020) report and the effects of lipid sources and replacement/inclusion levels variability on their reproductive performances, such as fecundity, fertilization rate, hatching rate, and larval survival. In general, all broodstock species can utilize plant-sourced lipid inclusion in their diet. *Cyprinus carpio* female broodstock can tolerate fish oil replacement levels in the range of 3-15% (Xu et al., 2016; Yeasmin et al., 2018); whereas the female koi variety can tolerate a range of 7.5-9.5% replacement levels without compromising its reproductive performance (Harshavardhan et al., 2021). The *Channa striatus, Oreochromis niloticus,* and *Oncorhynchus mykiss* female broodstocks benefit from 18.0%, 9.7%, and 12.9% lipid levels, respectively, and can achieve high fertilization rates of 78.5-91.7% and hatching rate of 74.4-89.6% (Ghaedi et al., 2016; Hajizadeh and Shinn, 2016; Noori et al., 2019).

**Table 4:** Summary of the effects of lipid sources and their replacement/inclusion levels on reproductive parameters of selected aquaculture freshwater species listed in FAO (2020) report.

**Fish species**	**Lipid source**	**Lipid replacement/ inclusion levels**	**Fecundity**	**Fertilization rate (%)**	**Hatching rate (%)**	**Larval survival (%)**	**Reference**
African catfish (*Channa striatus*)	SO, FO	18.0%	32,625		74.4	86.0	Ghaedi et al. (2016)
Nile tilapia (*Oreochromis niloticus)*	CLO, PO	9.7%	823.3	78.5	60.1		Hajizadeh and Shinn (2016)
Rainbow trout (*Oncorhynchus mykiss*)	PO, FO, CAO, CCO, CO, LNO, OLO, SFO	12.9%	13,200	91.7	89.6		Noori et al. (2019)
Common carp (*Cyprinus carpio)*	SFO, FO	3-5%	125,000	73.40			Xu et al. (2016)
Common carp (*Cyprinus carpio)*	OC	15.0%	355,963	84.00	87.33		Yeasmin et al. (2018)
Fancy koi, (*Cyprinus carpio var. koi)*	FO, PO, GNO, VGO	7.5-9.5%	120,522	58.33	51.38	26.28	Harshavardhan et al., (2021)

The values in the table show the studied groups that performed the best overall. Lipid replacement/inclusion levels presented are the optimum from reported studies. CO – corn oil, CLO – cod liver oil, CAO – canola oil, CCO – coconut oil, FO – fish oil, GNO – groundnut oil, LNO – linseed oil, OC – oil cake, OLO – olive oil, PO – palm oil, SO – soybean oil, SFO –sunflower oil, VGO – vegetable oil.

Lipids are retained in the muscle and liver in fish, mobilized during gametogenesis, transferred to the ovaries, and introduced into the egg/yolk as nutritional material, acting as the primary food source for the future embryo (Ma et al., 2020). Low levels of lipids and fatty acids in diets have been found to have adverse effects on reproduction and larval survival in various fish species (Du et al., 2018). Reproductive ability and egg quality are highly linked to the availability of dietary docosahexaenoic acid (DHA), eicosapentaenoic acid (EPA), and arachidonic acid (ARA), and their proportions in fish diets (Nguyen et al., 2010; Zakeri et al., 2011; Ghaedi et al., 2016; Nzohabonayo et al., 2017; Kabir et al., 2011).

The nutritional composition of lipids and fatty acids in the diets of *P. hypophthalmus* is believed to have a significant impact on reproductive development, including spawning performance, larvae supply, and egg quality (Zakeri et al., 2011; Ghaedi et al., 2016; Nzohabonayo et al., 2017; Noori et al., 2019). Fish reproductive hormone profiles are said to be improved by lipids and fatty acids (Izquierdo et al., 2001; Sattang et al., 2021). In comparison to the control diet, fish oil supplementation at 1% and 2% in the diet of hybrid catfish *(P. larnaudii x P. hypophthalmus)* showed higher levels of steroid hormones (Sattang et al., 2021). Similarly, varying dietary lipid levels have been shown to influence *P. hypophthalmus* spawning performance and egg quality. In their study, Kabir et al. (2019) found that diets with high lipid content (90-120 g kg^−1^) had superior results in gonadosomatic index (GSI), fecundity, egg weight and diameter, and fertilisation rate when compared to diets with low lipid content (60 g kg^−1^). Kabir et al. (2019) reported a higher success rate of spawning compared to the average success (22 – 42%) of *P. hypophthalmus* reported in Vietnam (Bui et al., 2010), where the comparably low spawning success recorded by hatchery producers might be linked to low-fat (40 – 60 g kg^−1^ lipid) diets reportedly used for broodstock development. An increase in egg mass and diameter of *P. hypophthalmus* related to lipid dietary intake could be ascribed to higher yolk content resulting in larger-sized eggs, which allow hatched larvae to survive for a longer period without food.

### 5.3 Carbohydrates

Carbohydrates are the most common energy-producing nutrients and are commercially essential in the formulation of fish feed. Carbohydrates like starch, which undergoes gelatinization during extrusion, are also required to make pellets with desirable floating characteristics (Kannadhason et al., 2011; Ishak et al., 2022). The use of dietary carbohydrates depends on the fish's feeding habits, environmental conditions, life stage, and the type of carbohydrate, among other factors (Honorato et al., 2010; Moro et al., 2016). Although there are few published studies on the quantitative and qualitative requirements for carbohydrates in Pangasius catfish and many farmed fish species, carbohydrates are included in fish diets due to the critical role it plays in preventing the catabolism of protein and lipids for energy needs (McGoogan and Gatlin, 2000), as well as the fact that dietary carbohydrates cause relatively few environmental problems.

### 5.4 Vitamins

Vitamins, which are required in modest amounts for normal growth, metabolism, health, and reproduction, are frequently not synthesised by fish. Water-soluble vitamins like vitamins B and C (ascorbic acid) as well as fat-soluble vitamins like vitamins A, D, and E are important in fish diets. Deficiency of vitamins in fish diets can cause stunted development, as well as abnormalities in colour and reproduction (Shahkar et al.,2015). Vitamins A, E, and C are the most studied vitamins in fish. (Oliva-Tales, 2012; Henry et al., 2020). Vitamin needs vary even within species depending on factors such as diet, age, physiological state, and the structure and function of their digestive system which collectively influence the ability of the fish to absorb, transport, and metabolize dietary vitamins (Volkoff & London,2018).

Vitamin E has been demonstrated to enhance the quality of gonads, fecundity, egg quality, embryonic development, percentage of fertilisation, hatching, and survival of larvae in studies on herbivorous/omnivorous (seabream, salmon) and carnivorous (carp, ayu) fishes as reviewed by Izquierdo et al., (2001). Deficiency of vitamin E in the diets of some fish species resulted in immature gonads, low fecundity, and fertility, reduced hatching rates, and fry survival. *P. hypophthalmus* broodstock were fed with different levels of vitamin E in their diet, ranging from 28.08, 146.65, 189.65, or 251.80 mg/kg respectively. The vitamin E and lipid contents of the eggs increased as the vitamin E dosage increased together with an improvement in gonadosomatic index, fecundity, egg diameter, hatching rate, reduction in the number of abnormal larvae, and the total number of larvae produced according to Yulfiperius et al. (2003). The best results were obtained with a diet containing 189.65 mg VE/kg, which significantly improved the hatching rate (78.77%), the total number of larvae (332,339/kg of broodstock), and abnormal larvae (0.19%). Hatching rates for other levels of vitamin E were 28.08 (40.81%), 146.55 (69.47%), and 251.80 (33.38%) respectively. This suggests that 189.65 mg kg^−1^ levels of vitamin E might be appropriate for the improved reproductive performance of pangasius catfish. In a similar vein, pindani (*Pseudotropheus socolofi* Johnson, 1974) fed dietary α-tocopherol demonstrated a significant improvement in egg sizes, fertilisation, hatching rates, number of spawns, batch fecundity, and relative fecundities per female or per g female as well as pre and post larval survival at an inclusion level of 100 mg kg^−1^ compared to that of 50 mg kg^−1^ (Erdogan & Arslar, 2019).

Vitamin E is an active component in the formation of somatic and gonadic structures, it is, therefore, crucial in determining the quality of the eggs produced by fish broodstock (Fitriliyani, Siswanto & Luchas, 2022). Broodstock of kissing gouramy *Helostoma temminckii* fed artificial diets supplemented with glutathione and vitamin E fortifications with levels of 300 mg kg^−1^ - 700 mg kg^−1^ increase the gonadosomatic index (GSI), hepatosomatic index (HSI), higher fecundity and larger egg diameter compared with single glutathione treatment without combination with vitamin E (Fitriliyani, Siswanto & Luchas, 2022). Additionally, vitamin E serves as an antioxidant, limiting the oxidation of essential unsaturated fatty acids in cells that are later used during embryogenesis to improve reproductive success, as well as improve sperm quality and protect sperm cells from oxidation (Yulfiperius et al., 2003; El-Sayed & Izquierdo, 2021).

Vitamin C or ascorbic acid (AA) is an antioxidant as well as an active enhancer of gonad development, where gonads (ovary and testes) have high concentrations of AA (Dabrowski & Ciereszko, 2001). Male freshwater Japanese eel, *Anguilla japonica* fed a high AA diet (1686 mg kg-^1^) have the highest GSI value amongst treatments with the testes retaining the highest AA concentration compared to other tissues (Shahkar et al. 2015). Moreover, AA displayed bactericidal properties and improved haematological parameters in *A. japonica* juveniles (Ren et al., 2005; 2007). AA deficiency leads to low seminal plasma protein concentration, declining sperm volume, and motility in male rainbow trout, *O. mykiss* (Ciereszko et al., 1996). Similarly, female *O. mykiss* responded positively to the high levels of AA-supplemented diet (220, 440, 880 mg kg^−1^) and exhibited the highest AA concentration in its ovary and ovulated eggs corroborated by high fecundity and low embryo mortality (Blom & Dabrowski, 1995). High AA concentrations indicate that AA is actively transferred to the gonads and retained during reproductive activities, showing the importance of AA for both male and female fish reproduction. Consequently, AA supplementation in the diets of *pangasius* broodstock could also enhance their reproduction.

Retinoids are derivatives of vitamin A that play a key role in many biological processes in animals, including immunity, vision, reproduction, growth, and development (Fontagné-Dicharry et al., 2010). Symptoms of retinoid deficiency in fish diets include decreased growth, reduced feed intake, and bone deformities as well as haemorrhages in the eyes, fins, and skin, leading to high mortality rates (Fernández-Díaz et al., 2006; Yang et al., 2008). Vitamin A has also been shown to be teratogenic (negatively affecting larva development) in many fish species when higher levels are included in diets. Juvenile rainbow trout showed retarded growth, scoliosis, necrotic fins, and lordosis when they were fed diets that contained more than 2700IU g^−1^ of vitamin A in diets (Fontagné-Dicharry et al., 2010). This suggests that vitamins are required to improve the reproductive performance of broodstock, but it is important to optimize the intake of these vitamins and establish appropriate amounts for different fish species and life cycle stages to avoid the consequences of both vitamin deficiency and dietary overdose. There is no specific scientific report for *Pangasius catfish* regarding their ability to utilize vitamin A for growth and reproduction. Table 5 present the effects of vitamins on fish reproductive performance and the general well-being of different farmed fish species.

**Table 5:** A summary of the effects of vitamins on reproductive performance and well-being of different farmed fish species.

Species	Dietary vitamin levels		Description	Reference
	**Treatment**	**Optimum**		
Pindani (*P. socolofi*) (Broodstock)	VE 121.3−270.0 mg kg^−1^	219.3 mg kg^−1^	Reproductive performance, hatching rates, and larval survival increased with increasing dietary VE levels up to 219.3 mg kg^−1^, then leveled off or declined	Erdogan & Arslar, 2019
Kissing gourami *(Helostoma temminckii)* (broodstock)	VE 300-700 mg kg^−1^	300 -700 mg kg^−1^	Increase gonadosomatic index (GSI), hepatosomatic index (HSI), and higher fecundity and larger egg diameter	Fitriliyani & Luchas1, 2022
*P. hypophthalmus* (broodstock)	VE 28.08, 146.65, 189.65 mg kg^−1^	189.65 mg kg^−1^	Improvement in gonadosomatic index, fecundity, egg diameter, hatching rate, reduction in the number of abnormal larvae, and increased larvae number	Yulfiperius et al., 2003
Japanese eel, *Anguilla japonica* (Broodstock)	Vit C 32, 206,423, 840, 1686 mg kg^−1^	410.8 & 911.8 mg kg^−1^	Increase GSI, initiates the proliferation of Sg, changes in testes color, and increased collagen level	Shahkar et al. 2015
Japanese eel, *Anguilla japonica* (juveniles)	Vit C 32, 762 mg kg^−1^	762 mg kg^−1^	Improved blood chemistry and immunological parameters	Ren et al., 2007
Japanese eel, *Anguilla japonica* (juveniles)	Vit C 3, 10, 27,126, 645, and 3,135 mg kg^−1^	27 & 645 mg kg^−1^	Increase growth, increased, high bactericidal activity, hematocrit, hemoglobin, total serum protein value, and liver and brain vitamin C concentrations.	Ren et al., 2005
Rainbow trout, Oncorhynchus mykiss (Broodstock)	Vit C 0, 30, 110, 220, 440 and 870 mg kg^−1^	440 & 870 mg kg^−1^	Lower levels of Vit C bellow the optimum lead to low seminal plasma protein concentration, declining sperm volume, and motility	Ciereszko et al., 1996
Rainbow trout, Oncorhynchus mykiss (Broodstock)	Vit C 0, 30, 110, 220, 440 and 880 mg kg^−1^	220, 440 & 880 mg kg^−1^	The highest AA concentration in its ovary and ovulated eggs are corroborated by high fecundity and low embryo mortality	Blom & Dabrowski, 1995
Rainbow trout (*Oncorhynchus mykiss*)	Vit A 0, 20, 700 or 0, 20, 200 IU/g	60- 200 IU/g	Females fed diet 200 showed better fecundity and increased growth. Females fed 700 showed a decline in survival from the eyed stage	Fontagné-Dicharry et al., 2010

Abbreviations: VE, vitamin E, Vit C, vitamin C, Vit A vitamin A, Sg, spermatogonia

### 5.5 Minerals

Minerals are inorganic elements that are necessary for the body's normal functions. Micro-minerals such as copper, iron, chromium, iodine, zinc, and selenium are required in small quantities as components in enzyme and hormone systems, and macro -minerals such as sodium, chloride, potassium, and phosphorous are required in higher quantities in the diets of fish (Hosnedlova et al., 2017).

Phosphorus deficiency can cause decreased fecundity in female fish, decreasing hatchability rates and resulting in a high level of malformations in newly hatched larvae. Calcium is a vital micro-mineral because it has been stated to be useful in the activation process of eggs when they come in touch with water during fertilization. Micro- mineral deficiencies in diets do not influence growth or reproduction, but they can be harmful if fish are exposed for lengthy periods (Volkoff and Sydney, 2018; Wischhusen et al., 2019). Selenium (Se) supplementation has been shown to have no discernible effects on growth and reproduction when given to rainbow trout (*Oncorhynchus mykiss*) broodstock for six months (Wischhusen et al., 2019). These findings are consistent with zebrafish studies that found no significant difference in egg production or mating success between groups fed deficient (0.09mg Se kg^−1^) compared to replete (0.65mg Se kg^−1^) diets for a period of 78, 97, and 133 days respectively (Penglase et al., 2014a). It is clear from the reports above, that dietary selenium as a micro -mineral does not affect the reproductive performance of fish when deficient in a diet for a short period but needs to be incorporated in diets in recommended amounts as prolonged deficiencies might have a negative impact on the general wellbeing of the fish.

Zinc (Zn) is a key helper molecule that plays a role in numerous physiological processes in fish, including growth, skeletal muscle development, and immunity (Lall, 2002; Trushenski et al., 2006; Liang et al., 2012). Zinc regulates transcription factors, DNA, and protein synthesis, making it essential for animal development and homeostasis (Lee & Nam, 2017). Kazemi et al. (2020) used preliminary data to reveal that dietary zinc can improve sperm and seminal plasma quality parameters as well as reproductive performance in *O. mykiss* male broodstocks. Fertilization rate, eyed-stage rate, hatching rate, sperm motility duration, and spermatocrit levels in broodfish were significantly affected by a 16-week feeding experiment of sexually matured rainbow trout males with mineral zinc supplemented diets (Kazemi et al., 2020; Nazari et al., 2021). Jiang et al. (2016) reported that zinc-sulphate enriched meals impacted sperm motility metrics in blunt snout bream *(Megalobrama amblycephal)*. Zinc mineral supplementation in fish broodstock diets has not been studied in many fish species, including the *pangasius* catfish.

### 5.6 Probiotics / Prebiotics

Probiotics are bioactive, living microbial food/feed additives that improve digestion and, more importantly, the microflora of the gastrointestinal tract (GIT) in general, therefore increasing nutrition and disease resistance. Probiotics have been shown to promote disease resistance and immunity, nutrition and feed utilisation, reproduction and development, and gut architecture and function, among other things (Wanka et al., 2018). Essential qualities of desirable probiotics for aquaculture include the absence of plasmid-encoded antibiotic resistance genes, being a non-pathogenic microbe, and being resistant to bile salt and low pH (Merrifield et al., 2010). Lactic acid bacteria (e.g*., Lactobacillus spp., Pediococcus spp., Enterococcus spp*.) and *Bacillus spp.* are among the most common probiotics, and have been found to influence aquatic animal growth and nutrient utilisation, as well as their resistance to pathogenic bacteria (Ai et al., 2011; Burbank et al., 2011). Probiotic strains used as feed additives produce digestive enzymes that synthesize vital nutrients such as proteins and essential fatty acids, as well as help with feed utilisation and digestion, which improves the reproductive physiology and the energy requirement during spawning activities (Rohani et al., 2022). Supplementation of enzyme-producing probiotics increased digestive enzyme activity (lipase, proteases, and amylase) and feed utilisation in *P. pangasius* fingerlings (Debnath et al., 2005), common carp (*Cyprinus carpio*) larvae (Yanbo & Zirong, 2006; Suzer et al., 2008), gilthead sea bream (*Sparus aurata,* L.) larvae (Suzer et al., 2008). In connection with supplemental phytase the mineral status of *Pangasius* is also documented (Debnath et al., 2004). Certain microbes also boost larval performance; a combination of *lactobacillid* bacteria administered to fertilized zebrafish, *Danio rerio* eggs resulted in consistent improvements in growth, various developmental indices, and survival (Rohani et al., 2022).

Dietary probiotic supplementation on the reproductive performances of freshwater species has been investigated in guppy, *Poecilia reticulate*, Mexican molly, *Poecilia sphenops*, green swordtail, *Xiphophorus helleri* southern platyfish *Xiphophorus maculates*, and zebrafish as reviewed by Gioacchini et al., (2014). Feeding zebrafish experimental diets containing *Lb. rhamnosus* (at 106 CFU g–1) for 10 days, showed a significant daily number of ovulated eggs and hatching rates compared to control levels, starting from the second day of administration (Gioacchini et al. 2010a; 2011). Similarly, the administration of *Lb. rhamnosus* after 10 days to killifish breeders, indicated a significantly higher gonadosomatic index fecundity, hatching rate, embryo survival 15.0 ± 1.5, 204.3 ± 49.2, 79.2 ± 6.6, 181.9 ± 47.7 respectively in females fed the probiotic supplemented diet when compared with females fed the control diet 8.0 ± 1.2, 107.9 ± 36.1,75.7 ± 10.8, 92.8 ± 28.0 respectively (Lombardo *et al*. 2011). The effects of probiotics in broodstock of numerous fish taxa, particularly *Pangasidae,* have been sparsely studied.

A prebiotic is a non-digestible food component that benefits the host by promoting the growth and/or activity of one or a few bacteria in the gastrointestinal tract. Despite the potential health and performance benefits seen in different terrestrial species, prebiotics has got less attention in fish and shellfish production (Ganguly et al., 2013). Prebiotics have been studied in fish and shellfish for their effects on growth, feed conversion, spawning, gut microbiota, cell damage/morphology, resistance to pathogenic bacteria, and innate immune parameters such as alternative complement activity (ACH50), lysozyme activity, natural haemagglutination activity, respiratory burst, superoxide dismutase activity, and phagocytin activity (Ringø et al. 2010). The platy fish*, Xiphophorus maculatus,* gonadosomatic index, fry production, relative fecundity, and fry length improved significantly with the administration of dietary prebiotic Immunogen at a rate of 1.5% compared to 0%, 0.5%, and 1%. (Abasali & Mohamad, 2011). Consistently, Zebrafish, *Danio rerio*, fed 0.4% dietary prebiotic annan-oligosaccharide spawned after 8 weeks, but those fed 0% annan-oligosaccharide did not. The number of vitellogenic oocytes increased considerably in fish fed dietary prebiotics (Forsatkar et al., 2018). Given the number of published studies on dietary prebiotic utilisation in fish broodstock, further study is needed to fully exploit the favourable effects of prebiotics on fish reproductive performance. The reports obtained in this area can be used to develop studies in the formulation of pangasius cat-fish feed.

## 6 Molecular approach in broodstock nutrition

Understanding the internal mechanisms governing specific biological and physiological functions and changes is crucial for aquaculture manipulation and enhancement. The application of molecular techniques such as genomics, proteomics, transcriptomics, and metabolomics is gaining traction in all fields of aquaculture, including nutrition (Martin & Król, 2017; Mekuchi et al., 2017), disease and immunity (Nathan et al., 2021; Waiho et al., 2021), stress responses (Wen et al., 2019), and growth (Causey et al., 2019; Lin et al., 2019). This section will focus on relevant molecular techniques pertinent to broodstock nutrition, specifically its potential use in enhancing broodstock development of *pangasius* catfish species.

### 6.1 Genotyping

High-throughput genomic technologies such as next-generation sequencing (NGS) and microarrays enable researchers to investigate genetic variants such as single nucleotide polymorphisms (SNPs) and large structural changes in DNA. The availability of sufficient genomic data further enables the construction of genetic linkage maps (Feng et al., 2018; Waiho et al., 2019) and the implementation of genome-wide association studies (GWAS) that would allow researchers to associate DNA variants to phenotypes with traits of interest (Taranto et al., 2018; Ali et al., 2020). The results from polymorphic markers, linkage maps, and GWAS are essential in breeding programs of broodstock, including marker-assisted selection (MAS), genome selection, and genome editing (Kim et al., 2018). Initially, due to the lack of sufficient genetic information, Sriphairoja et al. (2007) were unable to obtain any Amplified Fragment Length Polymorphism (AFLP) of sex-specific markers in *Pangasianodon gigas* and *P. hypophthalmus*. By using NGS, Vo et al. (2018) identified 11,009 SNPs from 400 wild and farmed *P. hypothalamus,* of which two SNPs (SNP5, SNP9) specific to the Vietnam population were proposed to be used as a coding sequence for commercial traceability of *P. hypophthalmus.* Currently, the full genome of *P. hypophthalmus* was made available by Kim et al. (2018) and Gao et al. (2021). By anchoring to the channel catfish chromosomes (n = 29), Kim et al. (2018) validated the chromosome number of *P. hypophthalmus* to be n = 30. Gao et al. (2021) further identified a tandem triplication of fatty acid binding protein 1 gene (fabp1) that could be associated with high fat content. The full genome data of *P. hypophthalmus* will aid in the future fine mapping of its genetic linkage map and quantitative trait loci (QTL) to aid the trait selection process, including nutrition-related traits such as high-fat. Additionally, the full genome availability of *P. hypothalmus* serves as a critical reference for GWAS studies to identify SNPs associated with sexual maturation and gonadal development in *P. hypothalmus* and other species of the same genus. For example, a GWAS study using Atlantic salmons from the Cermaq Canada broodstock program was conducted to identify potential SNPs associated with growth and sexual maturation (Gutierrez et al., 2015). The discovery of specific markers associated with growth, and early and late sexual maturation, and the subsequent identification of corresponding candidate genes revealed potential pathways that might be related to these processes. In a subsequent GWAS study, Mohamed et al. (2019) revealed the association of 13 SNPs for freshwater maturation and 48 SNPs for marine maturation, highlighting the highly polygenetic nature of sexual maturation in Atlantic salmon. These results are essential during broodstock diet development and nutritional enhancement. In a separate study, genetic variants of Atlantic salmon with high muscle omega-3 traits were identified and subsequent marker association analysis revealed the potential association of DHA/DPA ratio with elovl2, a protein that is known to be involved in DPA to DHA conversion (Horn et al., 2020). A similar approach is feasible for species in the family *Pangasiidae* to improve conventional broodstock selection programs by identifying specific trait-related alleles that could be screened and introduced into control breeding lines (Gutierrez et al., 2015).

### 6.2 Gene expression and transcriptome analysis

Much has been done in nutritional genomics or nutrigenomics, especially in lipid metabolism research. A recent review on elongase and desaturase in aquatic organisms discusses the current status of these genes and their functions (Monroig et al., 2022). Thus far, only limited full-length genes related to fatty acid biosynthesis pathways such as putative Δ6 fatty acyl desaturases (fads) (Rasal et al., 2016) have been reported in the family *Pangasiidae*. Table 6 presented a summary of gene expression studies done in Pangasisus fish. Growth-associated genes are characterized such as fads2 that regulates the Δ6 destaurase enzyme, and growth hormone 1 (GH1) gene which is reponsible for bones and tissue growth (Rasal et al., 2016; Andra et al., 2024). Immune-associated genes such as complement (C3), interleukin-1β (IL-1β), Interferon 2a γ (IFN2a), Interferon 2b γ (IFN2)b, interferon-γ (IFN-γ) and a2 MHC class II integral membrane protein alpha chain 2 (MHCII) are also done to assess innate immune reponses (cellular defence, bactericidal activities, lymphocyte activation, phagocytosis).

**Table 6:** A summary of gene expression studies on growth- and immune-associated genes in Pangasius species.

Pangasius species	Gene	Dietary nutrient	Description	Reference
Striped catfish *(Pangasianodon hypophthalmus*)	fads2	α-linolenic 0 (control), 0.5, 1.0, 1.5 and 2%	Liver Δ6 desaturase gene expression is up-regulated in fish fed with experimental diets	Rasal et al., 2016
*Pangasius pangasius*	IL-1β & C3	*Pseudomonas aeruginosa* FARP72	IL-1β & C3 genes upregulated significantly in kidney of PA diet-fed challenged fish.	Hoque et al., 2022
Striped catfish *(Pangasianodon hypophthalmus*)	IL-1β, C3 & GH1	Organic acid fermented Black soldier fly (BSF: *Hermetia illucens)*	The relative gene expression of growth hormone (GH1) was similar among the groups (P < 0.05). No differences were found in the expression of complement (C3) and interleukin-1β (IL-1β), but transferrin expression was up-regulated.	Andra et al., 2024
Striped catfish (*Pangasianodon hypophthalmus*)	IL-1β, IFN2a, IFN2b, and MHCII	Rambutan (*Nephelium lappaceum L*.) peel powder	Up-regulation of IL-1β, IFN2a, IFN2b, and MHCII was displayed in fish fed RBP40.	Xuan et al., 2022
Striped catfish *(Pangasianodon hypophthalmus*)	IFN-γ	Fucoidan rich seaweed extract (FRSE) from Sargassum wightii	After challenge with *A. hydrophila*, serum lysozyme activity, phagocytic activity and expression of interferon-γ gene of different treatment groups were higher than the pre-challenge treatment groups	Prabu et al., 2016

With the fast progress in NGS, researchers are now probing into the transcriptome profiles of species subjected to specific conditions to characterise any genes of interest that are differentially expressed (Kemski et al., 2020; Waiho et al., 2020). Yet, unlike other species of the same order (*Siluriformes*) that are more widely available, such as those of the genus *Clarias* and *Ictalurus*, the available transcriptome studies of *Pangasiidae* species are limited to phylogenomic (Chakrabarty et al., 2017) and terrestrial adaptation (Ma et al., 2021) studies. Based on the whole-body transcriptome of rainbow trout alevins broodstock, maternal diet history did not have a significant effect on the gene response of progeny before first feeding (Lazzarotto et al., 2016), thereby opening possibilities for the incorporation of plant-based diets for fish species. In species such as Atlantic salmon and rainbow trout where nutritional programming (introduction of plant-based diets) during early development has been successful (Geurden et al., 2013; Balasubramanian et al., 2016; Clarkson et al., 2017), Vera et al. (2017) revealed that nutritional history significantly affected the gene expression in key pathways, including pyruvate metabolism, glycolysis, and fatty acid metabolism, and the potential molecular mechanisms of nutritional programming based on the comparative liver transcriptome profiles of Atlantic salmon challenged with a plant-based diet. To improve the broodstock quality of gilthead seabream, Xu et al. (2021) found that the selection of broodstock with high fads2 expression and subsequently combined with broodstock nutritional programming (fish oil replacement by rapeseed oil) resulted in the production of offspring with up-regulated fads2 expression and improved growth. However, when broodstock nutritional programming is not being conducted, the cholesterol biosynthesis pathway will be upregulated to maintain homeostasis and thus negatively impact growth, as evident in the intestinal transcriptome profiles of juvenile yellow perch (*Perca flavescens*) when exposed to soybean meal-based diets during first feeding (Kemski et al., 2020). Owing to the increasing economic importance of species in the family *Pangasiidae,* broodstock nutrition of *Pangasiidae c*ould also be enhanced by the incorporation of both the selection of broodstock with high expression of nutrition-related genes and nutrition programming. Transcriptome analysis could be further used to identify important pathways during such enhancement programs.

### 6.3 Genome editing

With the advent and development of full genome sequencing, genome editing technology is increasingly gaining momentum as an important tool to elucidate biological processes and enhance or treat specific traits. Current genome editing technologies include zinc finger nucleases (ZFN), transcription activator like effector nucleases (TALEN), and clustered regularly interspaced short palindromic repeats (CRISPR) (Lu et al., 2021). ZFN is an artificial endonuclease that can be engineered to target and cleave a specific genomic sequence, which could result in gene knockout or gene correction and addition, based on the subsequent cellular repair processes (Urnov et al., 2010). During the early stages of DNA recombination and genome sequencing, the use of ZFN in fish primarily focused on model organisms such as zebrafish (Ekker, 2008; Foley et al., 2009). To overcome the difficulties during the application of ZFN (Chandrasegaran, 2017), TALEN, and subsequently CRISPR/Cas9 were developed (Becker & Boch, 2021). TALEN and ZFN are of lower efficiency, time consuming, and laborious when compared with CRISPR/Cas9 (Mushtaq et al., 2018). CRISPR/Cas9 is technologically simpler than TALEN and ZFN, comparatively more robust, and of higher efficiency, thereby enabling researchers to adopt genome editing in their routine research (Zhang et al., 2019). CRISPR/Cas9 has been widely used in fish aquaculture to enhance traits such as disease resistance, improved growth, and enhanced fertility (Gratacap et al., 2019). Well-known species commonly subjected to CRISPR/Cas9 in the aquaculture sector include different salmon species (Wargelius, 2019), Nile tilapia (Yan et al., 2019), zebrafish (Hruscha & Schmid, 2015; Liu et al., 2019), and medaka (Watakabe et al., 2018; Seleit et al., 2021). Among species within the order *Siluriformes*, CRISPR/Cas9 technology has been used on channel catfish to knock in the alligator *cathelicidin* gene into a targeted noncoding region (Simora et al., 2020), knockout of immune-related toll/interleukin 1 receptor domain-containing adapter molecule (TICAM 1) and rhamnose binding lectin (RBL) genes (Elaswad et al., 2018), and knockout of muscle suppressor myostatin gene (Khalil et al., 2017). From a nutritional perspective, CRISPR/Cas9 has been used to demonstrate the involvement of *elovl2* in the in vivo synthesis of PUFAs, specifically docosahexaenoic acid in Atlantic salmon (Datsomor et al., 2019b). In addition, by comparing two CRISPR-mediated partial knockout salmons (Δ6abc/5Mt with mutations in Δ6fads2-a, Δ6fads2-b, Δ6fads2-c, and Δ5fads2, and Δ6bcMt with mutations in Δ6fads2-b and Δ6fads2-c) and wild type individuals, Datsomor et al. (2019a) show that Δ5 Fads2 and Δ6 Fads2 target 20:4n-3 and 18:3n-3/18:2n-6, respectively within the LC-PUFA biosynthesis pathway of Atlantic salmon. Although there is still a lack of study on the broodstock nutrition of *Pangasiidae* species, the successful application of genome editing technologies in other fish species implies the feasibility of such methods to be used in *Pangasiidae* broodstock selection and enhancement as well. CRISPR/Cas9 can be specifically used to characterise the contribution and role of critical genes in the regulation of germline formation and egg developmental competence (Bobe, 2015). For example, Cas9 mRNA and sgRNA targeting the tyrosinase gene in the large yellow croaker (*Larimichthys crocea*) were successfully microinjected into fertilized eggs, highlighting the potential use of CRISPR/Cas9 in genome editing of fishes, especially targeting the embryonic stages (Li et al., 2023). However, the use of CRISPR/Cas9 in pangasius breeding still warrants intensive fundamental research as CRISPR/Cas9 technologies are species and gene-specific, thereby requiring in depth knowledge for precise targeting.

## 7 Conclusion

This review highlights the importance of dietary and molecular strategies in optimizing reproductive performance in Pangasius catfish broodstock. Strategic nutrient composition and genetic tools play a crucial role in enhancing broodstock conditions and seed production. The article identifies a significant knowledge gap specific to Pangasius catfish, which may impede the development of aquaculture for this species. Addressing this gap is essential for the advancement of Pangasius aquaculture. The impact of nutrient manipulation on reproductive physiology is evident, and the review provides insights into how dietary nutrients can be manipulated to improve reproductive events and outcomes. The review suggests that further research into the application of genomics and proteomics in aquaculture could lead to breakthroughs in seed production and reproductive performance, contributing to the sustainability of Pangasius catfish aquaculture.

## Funding

This review paper did not receive any funding or grant.

## Ethics statement

Since the current study is a review article and involves no live animals, ethical approval was not sought.

## CRediT authorship contribution statement

**Donald Torsabo:** Writing – review & editing, Writing – original draft, Conceptualization. **Sairatul Dahlianis Ishak:** Writing – review & editing. **Noordiyana Mat Noordin:** Supervision, Resources, Project administration. **Khor Waiho:** Writing – original draft. **Ivan Chong Chu Koh:** Writing – review & editing, Supervision, Resources. **Muhammad Abduh Yazed:** Writing – review & editing, Writing – original draft. **Ambok Bolong Abol-Munafi:** Supervision, Resources, Project administration.

## Declaration of competing interest

All persons listed as authors have read and contributed to preparing the manuscript attest to the validity and legitimacy of the data and its interpretation, and agree to its submission to Aquaculture. The manuscript has not been published nor is being considered for publication elsewhere. We have read and understood your journal's policies, and we believe that neither the manuscript nor the study violates any of these. There are no conflicts of interest to declare.
